# Intracranial Ewing sarcoma: four pediatric examples

**DOI:** 10.1007/s00381-017-3684-7

**Published:** 2017-12-28

**Authors:** Michael J. Yang, Ros Whelan, Jennifer Madden, Jean M. Mulcahy Levy, B. K. Kleinschmidt-DeMasters, Todd C. Hankinson, Nicholas K. Foreman, Michael H. Handler

**Affiliations:** 1 Department of Neurosurgery, The University of Colorado School of Medicine, 12605 E. 16th Avenue, Aurora, CO 80045 USA; 2 Department of Pediatrics, The University of Colorado School of Medicine, 12605 E. 16th Avenue, Aurora, CO 80045 USA; 3 Department of Neurology, The University of Colorado School of Medicine, 12605 E. 16th Avenue, Aurora, CO 80045 USA; 4 Department of Pathology, The University of Colorado School of Medicine, 12605 E. 16th Avenue, Aurora, CO 80045 USA; 5 Morgan Adams Foundation Pediatric Brain Tumor Research Program, Children’s Hospital Colorado, 13123 E. 16th Avenue, Aurora, CO USA

**Keywords:** Intracranial Ewing sarcoma, *EWSR1* gene, Pediatric brain tumor, PNET

## Abstract

**Background:**

Ewing sarcoma typically arises in bone and is unrelated to intraparenchymal small blue cell embryonal central nervous system (CNS) tumors previously designated primitive neuroectodermal tumors (PNETs). When the CNS is impacted, it is usually secondary to local extension from either the epidural space, skull, or intracranial or spinal metastases. Primary examples within the cranial vault are rare, usually dural-based, and are largely case reports in the literature. We detail four pediatric patients with solitary, primary intracranial Ewing sarcoma, all manifesting the archetypal EWRS1 gene rearrangement that confirms diagnosis.

**Procedure:**

Neurosurgical Department records, spanning 21 years (1995–2016), were reviewed to identify patients. Demographics, clinical history, pathological/genetic features, and clinical course were retrieved from the medical record and personal files of the authors.

**Results:**

Four patients, one male and three females, age 5 to 16 years, were identified. One presented in extremis from a large lesion, two with soft tissue masses, and the fourth as an incidental finding after being involved in a motor vehicle collision. Three had clear bony involvement: a 10-year-old girl with a large left temporal lesion had clear origin in the skull, with spiculated calcified striations throughout the mass; a 9-year-old girl presented with a bony left petrous apex mass; and a 16-year-old girl presented with a left temporal mass with extension to the dura and underlying bone erosion. Only the 5-year-old boy had a large left frontoparietal mass traversing the falx with no bony contact. All four tumors manifested the diagnostic *EWSR1* mutation and were treated with an Ewing sarcoma regimen. Outcomes were variable, with one patient showing progressive metastatic disease and death 3 years after presentation, one patient with disease-free survival 10.5 years after completion of therapy, and one alive and well at the completion of therapy 1 year after diagnosis. One patient completed therapy recently with post-therapy scans showing no evidence of disease.

**Conclusion:**

Testing for the *EWSR1* mutation confirms the diagnosis of Ewing sarcoma and excludes other types of embryonal CNS tumors. Long-term disease-free survival is possible with adherence to the appropriate therapeutic regimen after gross surgical resection.

## Introduction

Ewing sarcoma (EWS) is the second most common primary bone malignancy, arising mostly from long bones or the axial skeleton, including the pelvis, ribs, and vertebrae.^1^^,^ 2 Ewing sarcoma accounts for approximately 1% of all childhood malignancies, with approximately 200 new cases of Ewing sarcoma each year in the USA in adolescents under the age of 20 years.^3^^,^ 4 Ewing sarcoma can also be extraosseous.^1^

Primary intracranial Ewing sarcoma is exceedingly rare. It is considered a subtype of extraosseous EWS, given the most common origin is dura, and it is thought to account for only 1–4% of extraosseous EWS.^5^ Importantly, Ewing sarcoma differs from CNS embryonal tumors, formerly called central (supratentorial) primitive neuroectodermal tumors (cPNETs), with respect to underlying genetics, treatment, and prognosis.^5^^,^ 6 Given the rarity of primary intracranial Ewing sarcoma, particularly with respect to long-term outcome after therapy, we report our experience.

## Case reports

### Case 1

**Fig. 1:**
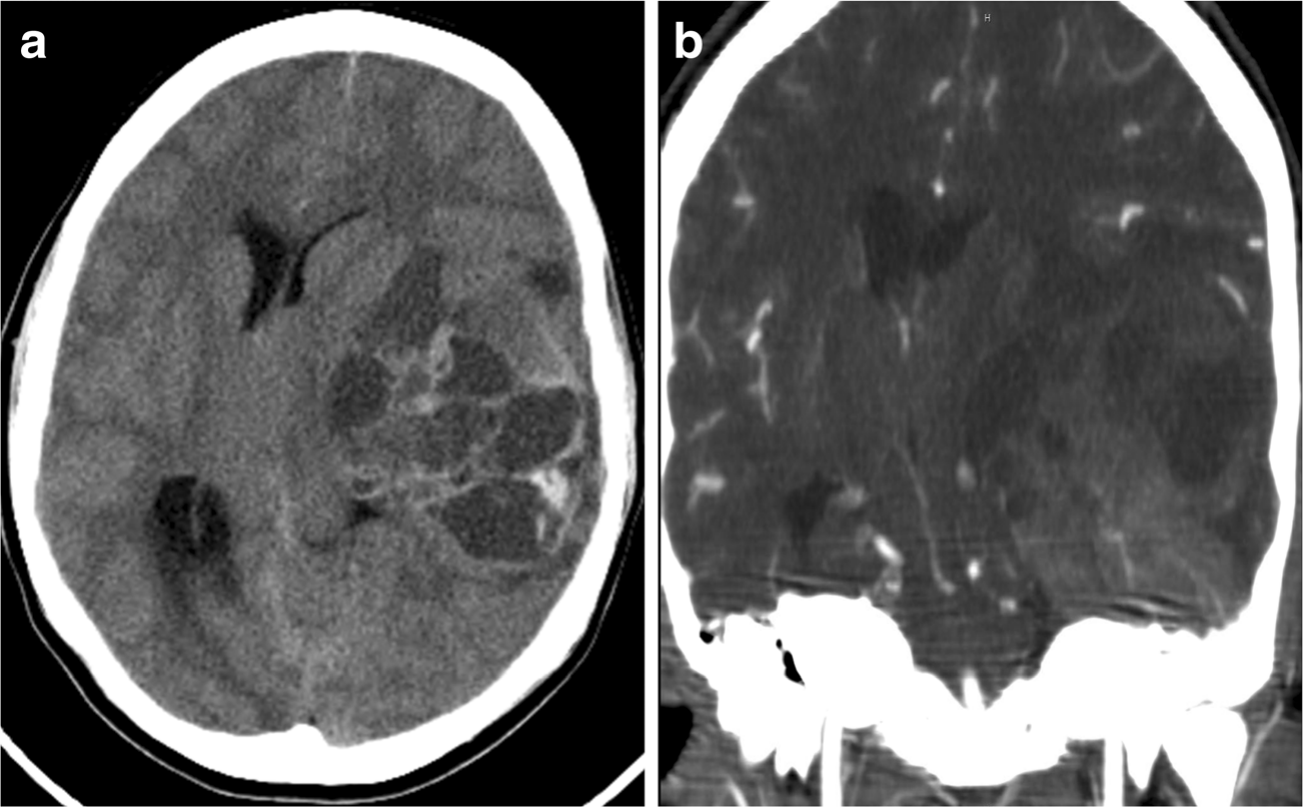
**a** Images from Case 1 of a 16-year-old female presenting with a left temporal lobe tumor. Non-contrast axial CT. **b** Post-contrast coronal CT demonstrating bony involvement along the intracranial surface of the inferior left temporal bone

A 16-year-old girl with a 2-week history of headaches and daily vomiting presented to the emergency department obtunded, with a fixed and dilated left pupil, and arousable only to pain. Computed tomography (CT) showed an intracranial left temporal mass exhibiting bone remodeling, indicative of probable bony origin (Fig. 1). She underwent emergent craniotomy for surgical resection of the lesion. Histological analysis demonstrated a small round blue cell tumor (Fig. 5a), and CD99 immunostaining was strongly positive (Fig. 5b). Polymerase chain reaction (PCR) testing detected *EWSR1* rearrangement diagnostic of EWS. Staging work-up, including a CT chest and whole body bone scan, demonstrated no evidence of extracranial disease. She was treated with ifosfamide/etoposide followed by doxorubicin/cyclophosphamide/vincristine for one cycle. She then received focal radiation with daily carboplatin. Following radiation, she received cyclophosphamide for stem cell harvest followed by vincristine/doxorubicin and ifosfamide/etoposide. She subsequently underwent triple autologous transplant with high-dose carboplatin/thiotepa. Complications of treatment included high-frequency hearing loss and mild memory issues and cognitive delay. She remains without evidence of tumor 10.5 years after her initial presentation.

### Case 2

**Fig. 2:**
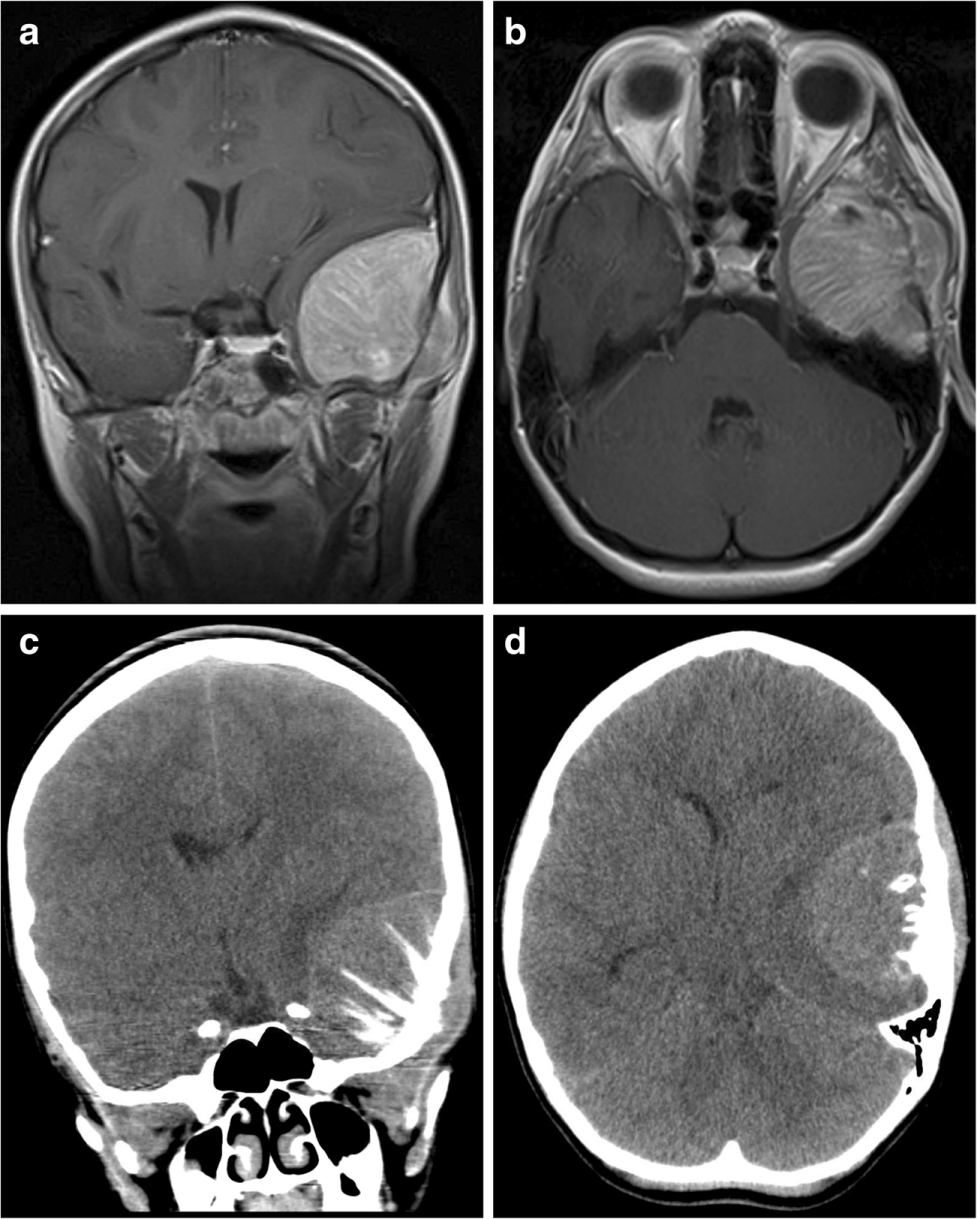
**a** Images from Case 2 of a 10-year-old female presenting with a left temporal lobe tumor. Extensive remodeling of the intracranial surface of the left temporal bone is observed. Coronal T1-weighted MRI with contrast. **b** Axial T1-weighted MRI with contrast. **c** Coronal non-contrast CT. **d** Axial non-contrast CT

A 10-year-old girl presented with a painful, palpable left temporal mass. She exhibited no neurological deficits nor signs or symptoms of systemic illness. Head CT demonstrated a spiculated tumor arising from the left temporal bone, resulting in substantial mass effect on the temporal lobe (Fig. 2). There was no evidence of an extracranial primary lesion. She underwent craniotomy for gross total resection of the lesion. Intraoperatively, the tumor was markedly adherent to dura but without intradural extension. Histological analysis demonstrated a small round blue cell tumor, with prominent CD99 staining of tumor cells. Fluorescence in situ hybridization (FISH) was positive for the *EWSR1* rearrangement diagnostic of EWS (Fig. 5c). The family initially pursued homeopathic treatments including nutritional support and tumor monitoring using serum anti-malignin antibody, a non-specific and non-sensitive marker of malignancy.^7^^,^ 8 The patient experienced a local recurrence at 3 months after presentation. Repeat complete resection of the tumor demonstrated intradural tumor extension although systemic work-up including chest CT and bone marrow biopsy remained negative for extracranial disease. She was treated according to the Children’s Oncology Group (COG) interval compression arm of AEWS0031 with a regimen of vincristine, doxorubicin, and cyclophosphamide alternating with ifosfamide/etoposide.^9^ She underwent focal radiation with a delay to concurrent chemotherapy due to an interval infection, and then completed continuation compressed therapy. Two years after her initial presentation, she was found to have local recurrence as well as bony metastases to the spine. She was subsequently treated with local irradiation, followed by complementary therapies which included high-dose IV vitamin C and a special diet high in whole fruits, vegetables, whole grains, and no processed sugar. Her disease progressed further with widespread bony dissemination, also treated with focal palliative radiation to the spinal metastases. Bone marrow biopsies at the time of each recurrence showed no evidence of malignancy. She died nearly 3 years after presentation.

### Case 3

**Fig. 3:**
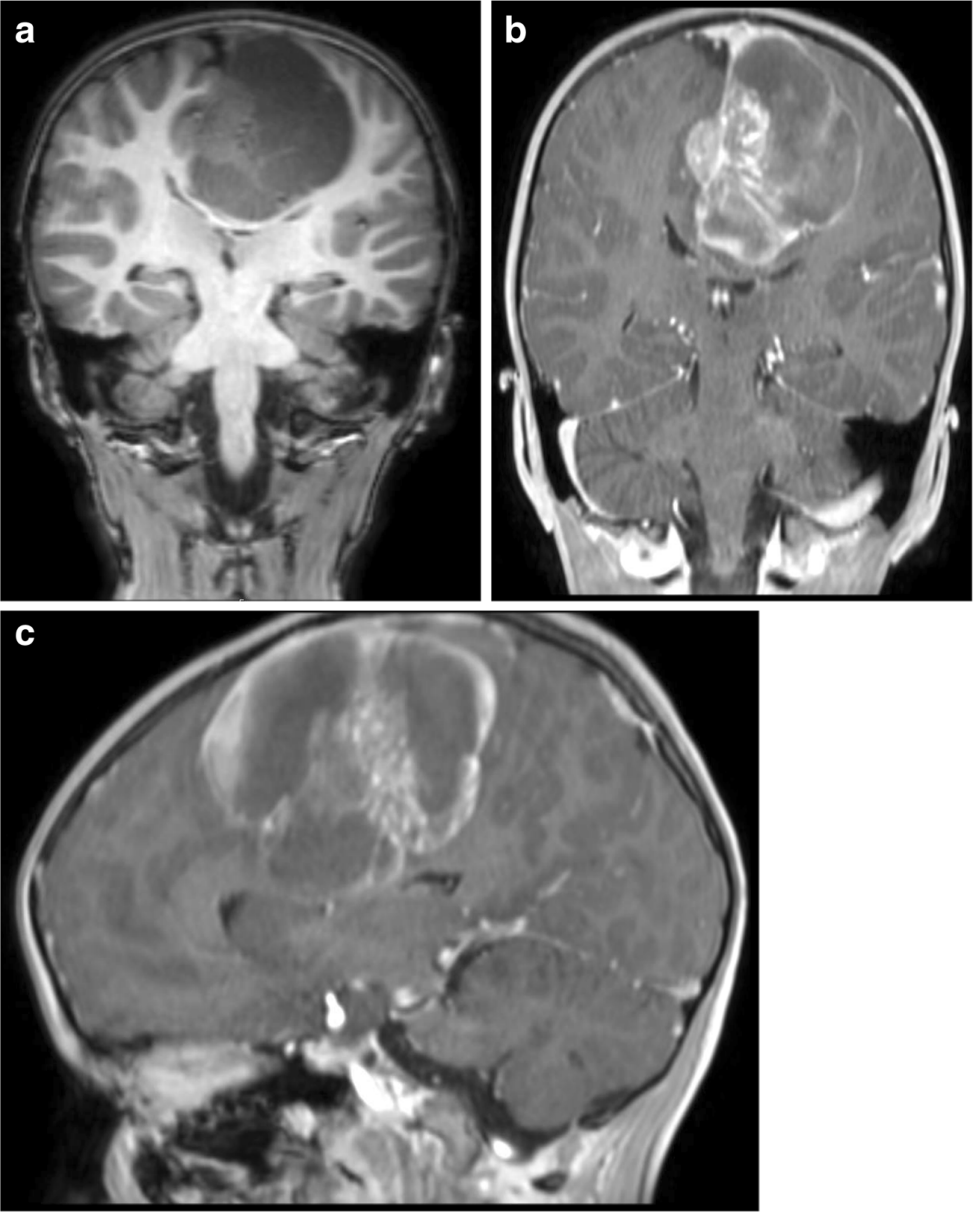
**a** Images from Case 3 of a 5-year-old male presenting with a left parietal lobe tumor with transfalcine extension. Coronal pre-contrast T1-weighted MRI. **b** Coronal post-contrast T1-weighted MRI. **c** Sagittal post-contrast T1-weighted MRI

A 5-year-old boy presented with 1-week duration of headaches progressing rapidly in frequency and severity. He also developed nausea, inattention, and impaired memory, followed shortly by gait instability and lethargy. Head CT showed a large multi-cystic parafalcine mass. Brain MRI demonstrated transfalcine extension, but there was no evidence of extracranial tumor (Fig. 3). He underwent endoscopic drainage of the cyst and a tumor biopsy with management of his immediate symptoms. Histology demonstrated a small round blue cell tumor. FISH showed the *EWSR1* rearrangement diagnostic for EWS (Fig. 5c). He subsequently underwent definitive resection of the tumor. He was treated according to the interval compression arm of AEWS0031 with focal radiation.^9^ He is currently asymptomatic with no evidence of disease progression 2 years after initial presentation.

### Case 4

**Fig. 4:**
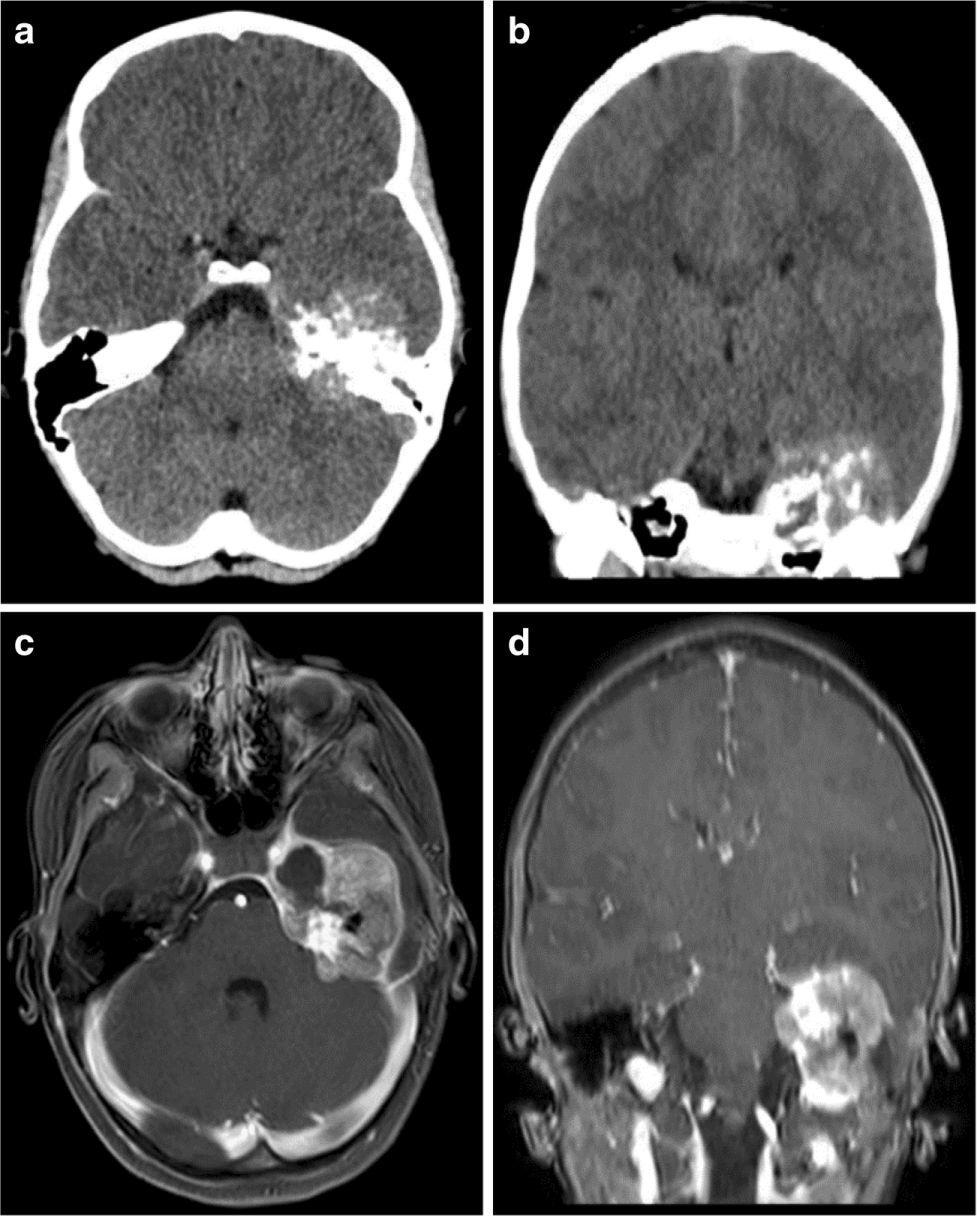
**a** Images from Case 4 of a 9-year-old female presenting with a left petrous apex tumor. Axial non-contrast CT. **b** Coronal non-contrast CT. **c** Axial T1-weighted post-contrast MRI. **d** Coronal T1-weighted post-contrast MRI

**Fig. 5:**
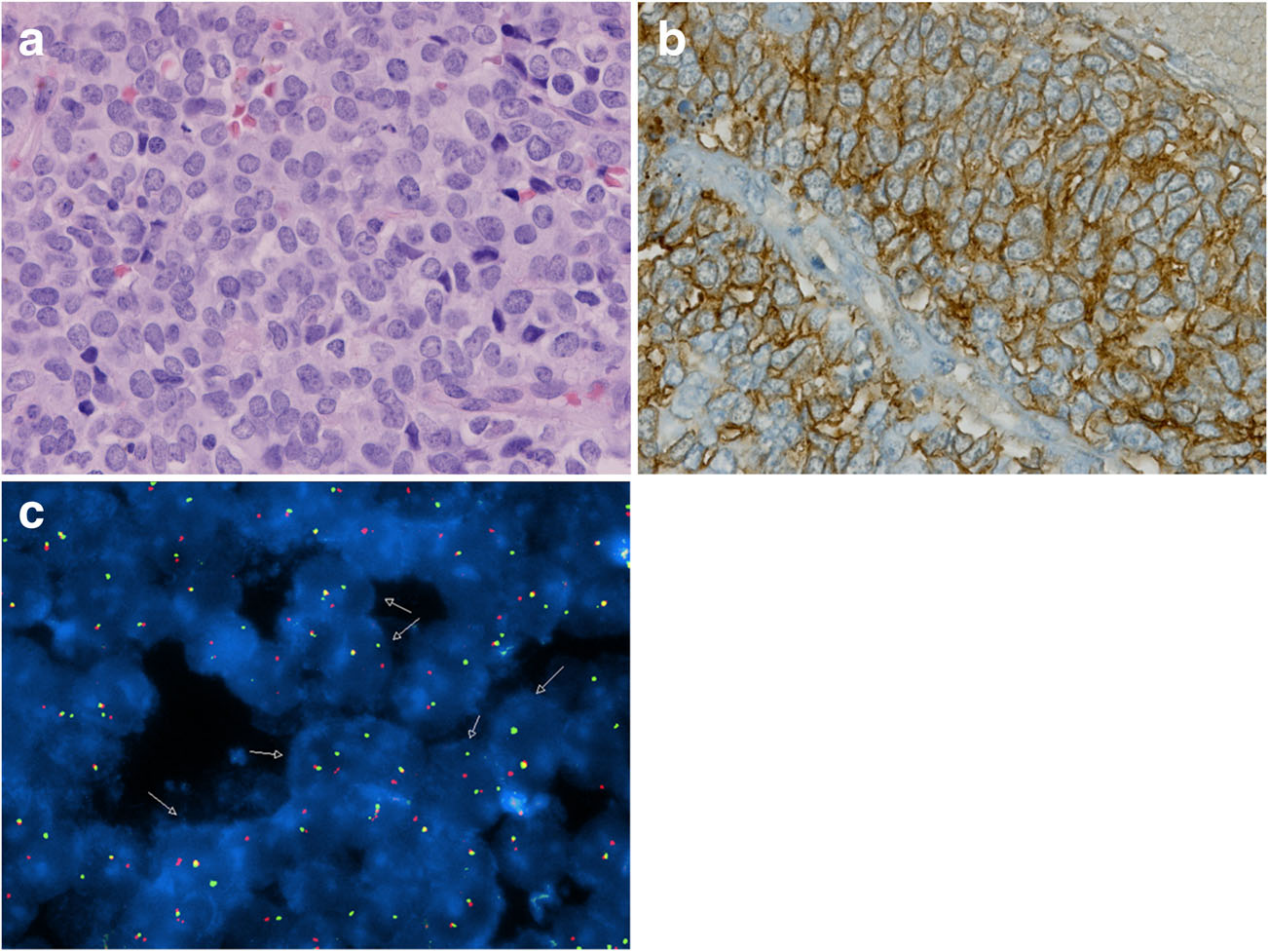
**a** H&E staining showing small blue cell tumor with scant cytoplasm and evident mitoses. **b** Immunohistochemical stain for CD99 showing strong expression in cell membranes. **c** FISH showing *EWSR1* rearrangement

A 9-year-old girl presented to an outside hospital with persistent headaches, nausea, and vomiting 2 days after a motor vehicle collision. A CT scan revealed a mass centered on the left petrous apex that demonstrated a mixed density lesion with calcifications and bony destruction, extending into the middle and posterior cranial fossae (Fig. 4). A brain MRI demonstrated an extra-axial mass with heterogeneous enhancement as well as some surrounding dural enhancement (Fig. 4). Upon tumor biopsy, pathology revealed a small round cell tumor with FISH testing demonstrating rearrangement of the *ESWR1* locus, confirming the diagnosis of EWS (Fig. 5c). The tumor was also strongly CD99 positive (Fig. 5b). She developed a left facial palsy that improved with dexamethasone treatment after biopsy. Metastatic work-up including chest CT, bone marrow biopsy, and whole body PET CT was negative. The patient was diagnosed and treated following the final results of AEWS0331 which defined the new standard of care for Ewing sarcoma to be the interval compression regimen outlined for Case #2 and #3. This patient was therefore treated with the now standard of care interval compression arm of AEWS1221 and is currently asymptomatic 1 year after presentation.

## Discussion

Ewing sarcoma is a rare childhood tumor, accounting for 1% of all pediatric malignancies, that typically presents in males ages 5 to 13 years.^3^^,^ 10 ^,^ 11 EWS is the second most common type of primary bone cancers, arising chiefly from long bones or the axial skeleton.^1^^,^ 2 Extraosseous EWS is less common, and primary intracranial EWS—whether dural or osseous in origin—is exceptionally rare.^2^

Although EWS is a type of “small round blue cell tumor,” it is unrelated to medulloblastoma, CNS embryonal tumors, or embryonal tumors with multilayered rosettes, C19MC altered.^1^ Because therapy for EWS differs considerably from embryonal CNS tumors, correct diagnosis is critical and typically involves documentation of the characteristic gene rearrangement.

Genetically, EWS is characterized by a translocation of the *EWS* gene, located on chromosome 22q12.^12^ While translocations involving a number of genes can be identified in EWS, the most common involves the *FLI1* gene located on 11q24, which creates a t(11;22)(q24;q12) translocation. This *FLI1* translocation, present in 90–95% of EWS cases 13, creates the *EWSR1* gene, which is an *EWS*-*FLI1* fusion; the resulting protein is a known aberrant transcription factor that interacts with *TP53* and *p21*.^12^^,^ 13 Downstream effects of the *EWS-FLI1* fusion gene include dysregulation of cell proliferation, differentiation, apoptosis, angiogenesis, invasion, and metastasis.^14^ By utilizing either fluorescence in situ hybridization (FISH) or reverse transcriptase polymerase chain reaction (rtPCR), it is possible to detect translocations diagnostic for EWS, specifically the *EWSR1* rearrangement, with 91–100% sensitivity and specificity.^15^ Within our series of four cases, all four patients exhibited the *EWSR1* rearrangement.

Overexpression of CD99, a cell surface glycoprotein and a product of the *MIC2* gene, is seen in essentially all cases of EWS.^16^ Positive CD99 immunohistochemical staining on cell membranes is a highly sensitive marker of EWS, although it is not highly specific, being seen in other CNS tumors including lymphoblastic lymphomas, ependymomas, and rhabdomyosarcomas.^17^ In contrast with EWS, however, CNS embryonal tumors do not express the *MIC2* gene, and thus will not positively stain for CD99.^18^ Of the four cases in our series, tumors from three patients exhibited strongly positive staining for CD99, which further supports the diagnosis of primary intracranial Ewing sarcoma.

Treatment of EWS includes maximal surgical resection with aggressive chemotherapy and focal radiation.^19^ In systemic EWS, neoadjuvant chemotherapy has also been shown to improve cytoreduction and achieve local control of tumors prior to surgical resection; it also has the benefit to assess tumor response to chemotherapy.^20^ Current first-line chemotherapy for EWS includes vincristine, doxorubicin, and cyclophosphamide, alternating with ifosfamide and etoposide.^21^ The completed COG AEWS0031 protocol improved clinical outcomes using interval-compressed chemotherapy; this has now become the standard of care therapy for EWS in addition to local control with maximal surgical resection and focal radiation.^9^ EWS usually requires only focal radiation (unless dissemination is present at diagnosis), unlike CNS embryonal tumors, such as medulloblastoma, that receive full craniospinal irradiation plus a boost to the tumor bed.^6^

Three of the four patients in our series received initial maximal surgical resection, followed by the either standard of care interval-compressed chemotherapy (Case #2, #3, and #4) or high-dose chemotherapy with autologous stem cell transplant (Case #1) and focal radiation. Two of these patients have had one and 10.5-year disease-free survival, respectively. The third patient pursued alternative therapies including homeopathy, diet, and IV vitamin C in addition to interval-compressed chemotherapy and succumbed to her illness after relapsing with widespread extracranial bony metastasis. The final patient has completed therapy and end of therapy scans demonstrated no evidence of disease. This patient did not undergo gross total resection as it was felt that total resection at the petrous bone was unlikely and the surgical risk was not justified.

Previous case studies of primary EWS involving the central nervous system have been reported.^2^^,^ 5 ^,^ 6 ^,^ 17 Srivastava et al. described extraosseous primary intracranial EWS occurring in the cavernous sinus.^17^ Primary EWS of the petroclival bone was reported by Balasubramanian et al.^2^ Navarro et al. detailed a 3-year-old boy with primary intracranial EWS of the tentorium who presented with intracranial hemorrhage.^6^ Upon literature review, Navarro et al. found that 41% of 17 patients with intracranial EWS presented with tumoral hemorrhage, and all but one of those tumors were found to have meningeal/dural origin.^6^

Salunke et al. examined a series of 10 cases of primary osseous intracranial EWS, six of which occurred in pediatric patients.^5^ Four involved the temporal bone, two the frontal bone, two the sphenoid bone, one the occipital bone, and one the ethmoid bone. In only one of 10 cases was primary intracranial EWS found to have metastasized at the time of presentation. The prognosis of primary intracranial EWS was found to be better than that of peripheral EWS, with a 5-year survival rate ranging from 39 to 65% for primary intracranial EWS.^5^

Within our case series, none of the four patients presenting with primary intracranial EWS had metastases at time of diagnosis. However, one patient did develop metastases during her third relapse following interval compression chemotherapy, re-irradiation, and high-dose vitamin C therapy. Two patients have shown disease-free survival (one at 1 year, the other at 10.5 years) and one patient is too early in her post-therapy monitoring to assess outcome, although end of therapy scans demonstrate no signs of disease.

Our case series is small with some patients still relatively close to completion of therapy making it difficult to make recommendations for therapy based on this experience, but important considerations for therapy can be gleaned from larger studies of extracranial EWS. As discussed above, large collaborative studies have shown that interval-compressed chemotherapy results in improved survival outcomes and is now standard of care.^9^ Less aggressive surgical approaches may be required in some cases of intracranial EWS, as demonstrated in Case #4, where the risk associated with total resection of the petrous bone was not justified. This could result in decreased survival for these patients. A review of outcomes for EWS in the National Cancer Database found a decreased 5-year survival in patients with radiation alone (52.5%) compared to patients treated with surgery alone (77.2%).^22^ A further analysis of patients treated in a Brazilian collaborative study also found that patients treated with radiation alone for local control had considerably worse outcomes with only a 17.8% survival at 5 years when treated with radiation alone.^23^ These results should be considered when evaluating the potential for complete resection of intracranial lesions and consideration for more aggressive surgery including the risks and benefits of cranioplasty.

With an increased understanding of the molecular diagnosis of CNS tumors, the importance of molecular studies for the definitive diagnosis of tumors is becoming more apparent. This has resulted in new classifications as outlined by the WHO Classification of Tumors of the Central Nervous System.^24^ For EWS specifically, an integrated genomic analysis of 323 tumors previously diagnosed as cPNET found 2% of those patients were subsequently molecularly identified as EWS. While this would have a consideration for surgical approaches as noted above, it would also significantly affect chemotherapy choice and potential relapse therapies.^25^

In conclusion, Ewing sarcoma typically presents as a PNET most commonly arising from the long bones or axial skeleton. Primary intracranial Ewing sarcoma is extremely rare and can easily be mistaken for CNS embryonal tumors. The cytogenetics, treatment regimen, and disease-free survival vary considerably between primary intracranial EWS and other more commonly seen CNS embryonal tumors. All supratentorial small round blue cell tumors should be tested for the *EWSR1* gene rearrangement for accurate diagnosis, to ensure all patients are started on the most appropriate treatment to optimize outcomes.
